# Novel Thyroid Hormone Receptor β Gene Variant (F245L) Causing Mild Resistance to Thyroid Hormone

**DOI:** 10.1210/jcemcr/luaf267

**Published:** 2025-11-19

**Authors:** Keiko Yamagami, Naotetsu Kanamoto, Yui Yamashita, Yumiko Sasai, Tetsuya Tagami

**Affiliations:** Department of Endocrinology, Osaka City General Hospital, Osaka 534-0021, Japan; Department of Endocrinology, Osaka City General Hospital, Osaka 534-0021, Japan; Department of Endocrinology, Osaka City General Hospital, Osaka 534-0021, Japan; Department of Endocrinology, Osaka City General Hospital, Osaka 534-0021, Japan; Division of Endocrinology and Metabolism, Clinical Research Institute, National Hospital Organization Kyoto Medical Center, Kyoto 612-8555, Japan

**Keywords:** variant, resistance to thyroid hormone, thyroid hormone receptor beta gene

## Abstract

A novel thyroid hormone receptor β (*THRB*) variant was identified in a 34-year-old Japanese man with resistance to thyroid hormone β (RTHβ). He presented with mildly elevated serum thyroid hormone and nonsuppressed TSH levels in the absence of a goiter. Genetic testing revealed a novel heterozygous missense variant of *THRB* (c.733T > C; p.F245L). In vitro transient gene expression assays demonstrated that the F245L mutant receptor had only mildly reduced transcriptional activity at physiological and low triiodothyronine concentrations and did not exhibit apparent dominant-negative activity. The F245L variant is located within a known cluster of functionally important residues in the ligand-binding domain, adjacent to R243Q, R243W, and P247L, which are also associated with mild phenotypes. Although the patient had a Rathke cleft cyst, its size decreased over time, whereas the thyroid function remained unchanged, suggesting an independent occurrence of the 2 conditions. To the best of our knowledge, this is the first report to provide functional evidence of the pathogenicity of the F245L variant. This case highlights the importance of integrating clinical, biochemical, and molecular data to diagnose and characterize RTHβ, particularly in patients with subtle phenotypes and novel genetic variants.

## Introduction

Resistance to thyroid hormones (RTH) is a clinical syndrome characterized by impaired sensitivity to thyroid hormones. It is commonly caused by mutations in the thyroid hormone receptor (TR) β (*THRB*) gene [1]. Since the first report of a *THRB* missense variant causing RTHβ, 236 different variants have been identified in 805 families [1]. These variants are predominantly located in the functional areas of the ligand-binding domain and adjacent hinge region and are clustered in 3 regions within exons 7 through 10. Here, we report a case of RTHβ concurrent with Rathke cleft cyst (RCC) harboring a novel *THRB* variant and present functional analyses of this novel variant using transient gene expression experiments.

## Case Presentation

A 34-year-old Japanese man with an unremarkable birth and developmental history and no prior medical issues presented with a 5-year history of palpitations at rest, tremors, bilateral lower-limb numbness, and excessive sweating, all of which progressively worsened over the past year. Four months before presentation, the patient visited a primary care physician because of persistent lower-limb paresthesia, and abnormal thyroid function was identified, prompting a referral to our hospital for further evaluation. The patient had no known family history of thyroid disorders.

On physical examination, his height was 175.5 cm, weight was 75.5 kg, and body mass index was 24.5 kg/m². The vital signs included a regular pulse rate of 68 beats per minute, blood pressure of 131/84 mm Hg, and body temperature of 36.4 °C. No proptosis or goiter was noted. Mild tremor of the fingers was noted; however, no other significant physical findings were observed.

## Diagnostic Assessment

**Figure 1.**
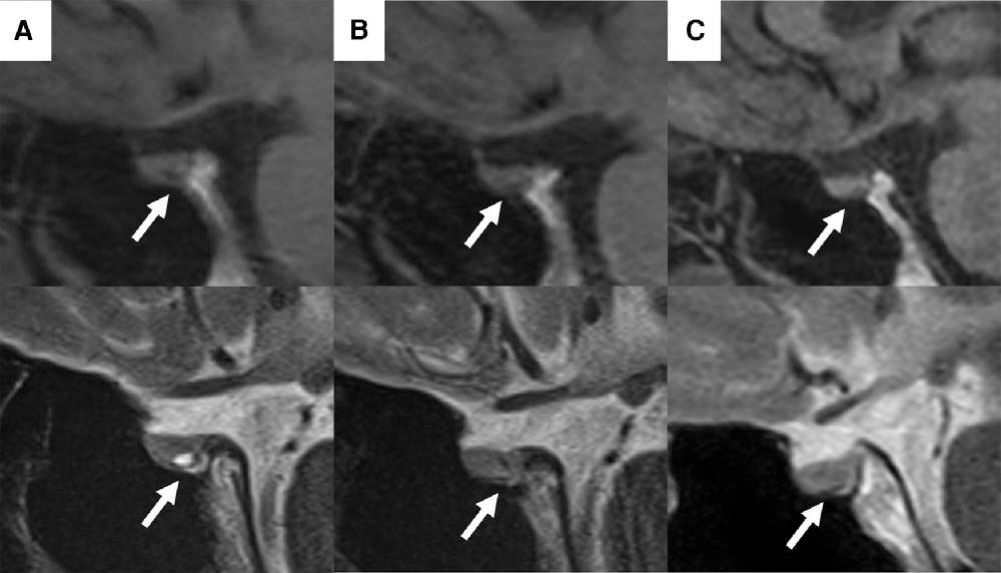
Sagittal T1-weighted (upper) and T2-weighted (lower) MRI scans of the pituitary gland. (A) At the time of referral. A small cystic lesion is visible in the anterior pituitary gland (arrow). (B) Three years after referral. The cystic lesion in the anterior pituitary gland has decreased in size (arrow). (C) Nine years after referral. The small cystic lesion in the anterior pituitary gland remains reduced in size (arrow).

The patient's thyroid function test (TFT) results using Elecsys (Roche Diagnostics, Mannheim, Germany) were as follows: free T4 (FT4), 2.4 ng/dL (30.9 pmol/L) (reference range, 0.9-1.7 ng/dL [11.6-21.9 pmol/L]); free T3 (FT3), 5.2 pg/mL (8.0 pmol/L) (reference range, 2.3-4.0 pg/mL [3.5-6.1 pmol/L]); and TSH, 2.66 μIU/mL (2.66 mIU/L) (reference range, 0.50-5.00 μIU/mL [0.50-5.00 mIU/L]). TFTs were repeated using a different platform (Architect, Abbott, Wiesbaden, Germany), and the results were concordant with those from the initial assay, demonstrating elevated FT4 and FT3 levels and nonsuppressed TSH levels. To further rule out assay interference, serum samples were reassessed after polyethylene glycol precipitation and protein A affinity column treatment; neither procedure altered the measured concentrations of TSH, FT4, or FT3. Antibodies against the TSH receptor (Elecsys, Roche Diagnostics) thyroglobulin (Architect, Abbott), and thyroperoxidase (Architect, Abbott), were negative. Thyroid ultrasonography revealed a normal thyroid volume and increased vascularization. Magnetic resonance imaging (MRI) of the pituitary gland revealed a small anterior cystic lesion, strongly suggestive of an RCC (Fig. 1A). The anterior pituitary hormone levels were within the reference range.

**Figure 2.**
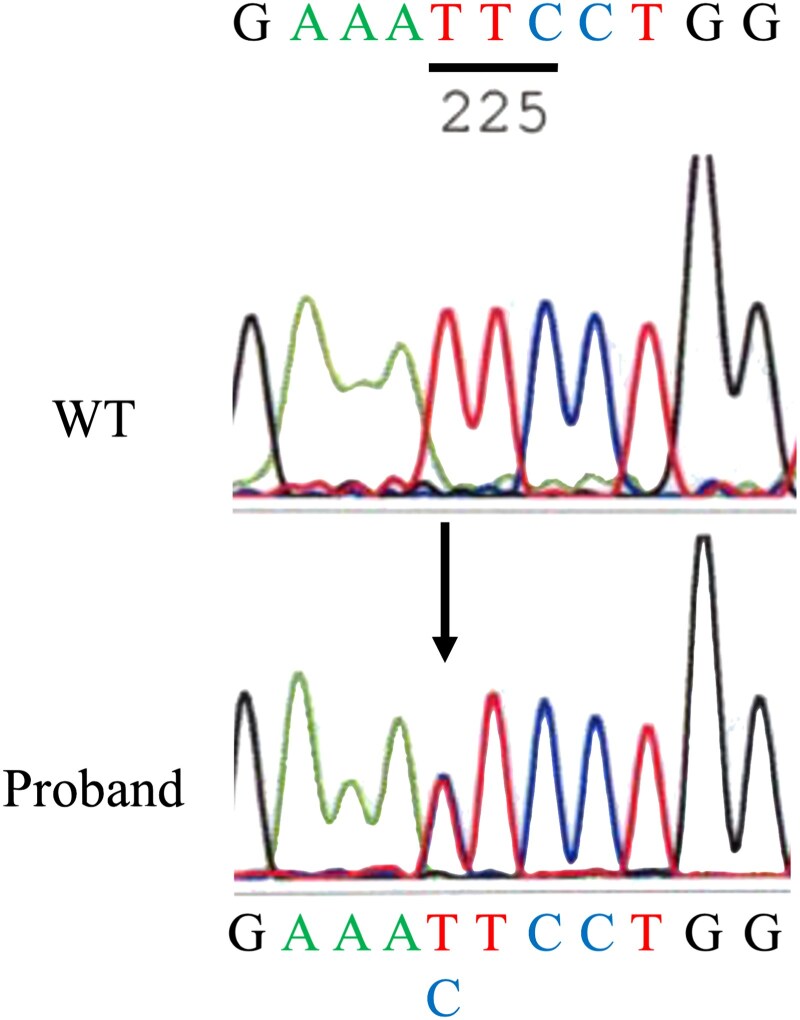
Genomic DNA sequence derived from white blood cells of the proband, covering the *TRHB* variant region. Genomic DNA was extracted from white blood cells using the QIAamp DNA Mini Kit (Qiagen, Tokyo, Japan) according to the manufacturer's instructions and amplified using polymerase chain reaction. Mutational analysis of *THRB* was performed by direct sequencing using an ABI PRISM 3130 (Applied Biosystems, Foster City, CA, USA). The chromatogram of the proband shows a single nucleotide substitution, a T to C transversion in exon 7 (c.733T > C, p.F245L). WT, wild-type.

TFT results revealed that thyroid hormone levels remained high and nonsuppressed, suggesting a diagnosis of RTH. Genetic testing was performed by direct sequencing of exons 7 through 10 of *THRB* (NM_000461.5 and NP_000452.2) and revealed a novel single nucleotide substitution (c.733T > C, Fig. 2), resulting in the replacement of normal phenylalanine at position 245 with leucine (p.F245L) in the proband. The variant allele was identified as GRCh38: 3:24143506 A > G and is registered in the ClinGen Allele Registry (CA351891315). This variant had not been reported previously in the Single Nucleotide Polymorphism Database, ClinVar, or gnomAD population database (releases r2.1.x, r3.1.x, and r4) [2], supporting its rarity; it has been newly registered in ClinVar (SCV006324311). In silico analyses predicted a deleterious effect, being classified as deleterious, damaging, and possibly damaging by PROVEAN [3], SIFT [4], and PolyPhen-2 [5], respectively. Consistently, prediction using AlphaMissense [6] also indicated a pathogenic effect, with a high pathogenicity score of 0.995. The patient's mother showed normal TFT results and did not express the variant. His father had divorced his mother and could not be contacted. He had no siblings, and other family members could not be examined. The patient's serum α glycoprotein subunit measurement, thyrotropin-releasing hormone stimulation, and the T3 suppression test were not performed because of insufficient biochemical evidence supporting the diagnosis and identification of the pathogenic variant by genetic testing. Written informed consent for genetic testing was obtained from both the patient and his mother.

**Figure 3.**
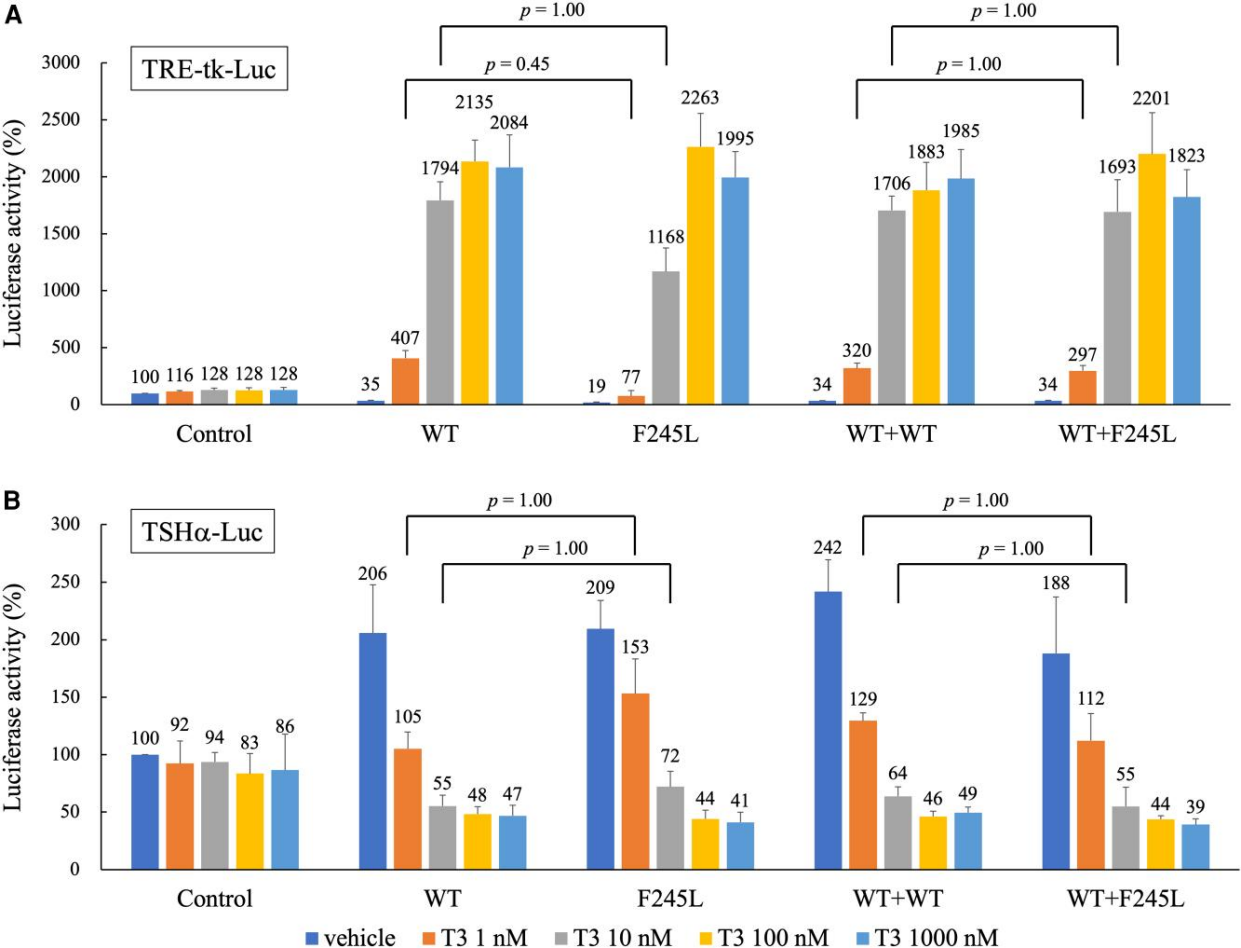
Function of the mutant thyroid receptor (TR). Fifty nanograms of each expression plasmid (wild-type [WT] or F245L), alone or in combination (WT + F245L), were transfected into TSA-201 cells together with 100 ng of TRE-tk-Luc (A) or TSHα-Luc (B). The total amount of plasmid DNA was kept constant by adding the corresponding amount of pCMX vector without a cDNA insert. Cells were incubated in the absence or presence of 1 to 1000 nM T3 and harvested for measurements of luciferase activity using a Dual-Luciferase Reporter Assay System (Promega, Madison, WI, USA). The transfection efficiencies were corrected for the internal control. Results are shown as means ± SD from at least 3 transfections performed in quadruplicate. Promoter activity is expressed relative to that of cotransfected with pCMX without a cDNA insert treated with vehicle. Statistical analyses of transcriptional activity among multiple groups were performed using the Kruskal-Wallis test, followed by pairwise Mann-Whitney *U* tests with Bonferroni correction for multiple comparisons. A *P* value < .05 was considered statistically significant. All analyses were conducted with EZR [9], which is a graphical user interface for R (The R Foundation for Statistical Computing, Vienna, Austria).

The functional characteristics of mutant TR were assessed in vitro using transient gene expression assays [7] with positively and negatively regulated promoters. Mutant TR (F245L) expression plasmids were generated by oligonucleotide-directed mutagenesis using the wild-type human TRβ1 expression plasmid, pCMX-hTRβ1, as a template and verified by sequencing. Using the positively regulated reporter plasmid containing 2 copies of a palindromic thyroid hormone response element upstream of the thymidine kinase promoter (tk109) in the pA3 luciferase vector, TRE-tk-Luc, and the negatively regulated reporter plasmid containing 846 bp of the 5′-flanking sequence and 44 bp of exon 1 from the human glycoprotein hormone α-subunit gene in the pA3 luciferase vector, TSHα-Luc [8], luciferase activity exhibited T3-dependent alterations following co-transfection with the wild-type TRβ1 expression plasmid. However, following co-transfection with a mutant TR expression plasmid, luciferase activity was altered only mildly in a dose-dependent manner at physiological (1 nM) and low (10 nM) T3 concentrations and showed no apparent alterations at high (100-1000 nM) T3 concentrations compared to that after co-transfection with the wild-type TRβ1 expression plasmid (Fig. 3A and 3B). To examine the dominant-negative activity of mutant TR, a mutant TR expression plasmid was co-transfected with the wild-type TRβ1 expression plasmid. The transcriptional activity of the positively and negatively regulated promoters showed no statistically significant differences [analyzed using EZR (Easy R) [9]] compared with that following transfection with the wild-type TRβ1 expression plasmid alone (Fig. 3A and 3B). These results indicate that no apparent dominant-negative effect was observed under the experimental conditions.

## Treatment

After the diagnosis of RTHβ was confirmed, treatment was initiated in the outpatient clinic with 5 mg bisoprolol for palpitations; however, the treatment was discontinued 4 months later because of the lack of improvement in the patient's symptoms. No other medications were used for treatment.

## Outcome and Follow-up

Although palpitations persisted, other subjective symptoms were controlled, and the patient was observed without treatment. The TFT results using Elecsys showed persistently elevated FT4 and FT3 levels, along with nonsuppressed TSH levels (Table 1). MRI scans of the pituitary gland showed that the small cystic lesion had decreased in size 3 years later and has remained stable without regrowth since then (Fig. 1B and 1C).

**Table 1.** Serial measurements of thyroid function during the clinical course

	Reference range	Initial visit	1 years	2 years	3 years	4 years	5 years	6 years	7 years	8 years	9 years	Current status
TSH	0.50-5.00 μIU/mL	2.66 μIU/mL	1.55 μIU/mL	1.34 μIU/mL	2.90 μIU/mL	1.76 μIU/mL	2.49 μIU/mL	1.13 μIU/mL	2.24 μIU/mL	1.02 μIU/mL	1.64 μIU/mL	1.98 μIU/mL
	(0.50-5.00 mIU/L)	(2.66 mIU/L)	(1.55 mIU/L)	(1.34 mIU/L)	(2.90 mIU/L)	(1.76 mIU/L)	(2.49 mIU/L)	(1.13 mIU/L)	(2.24 mIU/L)	(1.02 mIU/L)	(1.64 mIU/L)	(1.98 mIU/L)
FT4	0.9-1.7 ng/dL	2.4 ng/dL	2.0 ng/dL	2.0 ng/dL	2.1 ng/dL	2.3 ng/dL	2.2 ng/dL	2.3 ng/dL	2.3 ng/dL	1.9 ng/dL	2.4 ng/dL	2.1 ng/dL
	(11.6-21.9 pmol/L)	(30.9 pmol/L)	(25.7 pmol/L)	(25.7 pmol/L)	(27.0 pmol/L)	(29.6 pmol/L)	(28.3 pmol/L)	(29.6 pmol/L)	(29.6 pmol/L)	(24.5 pmol/L)	(30.9 pmol/L)	(27.0 pmol/L)
FT3	2.3-4.0 pg/mL	5.2 pg/mL	4.9 pg/mL	4.8 pg/mL	4.3 pg/mL	5.4 pg/mL	4.9 pg/mL	4.7 pg/mL	5.2 pg/mL	4.2 pg/mL	4.7 pg/mL	4.9 pg/mL
	(3.5-6.1 pmol/L)	(8.0 pmol/L)	(7.5 pmol/L)	(7.4 pmol/L)	(6.6 pmol/L)	(8.3 pmol/L)	(7.5 pmol/L)	(7.2 pmol/L)	(8.0 pmol/L)	(6.5 pmol/L)	(7.2 pmol/L)	(7.5 pmol/L)

Abbreviations: FT3, free T3; FT4, free T4.

## Discussion

We report a novel *THRB* variant located within one of the clusters of *THRB* between amino acid residues 234 and 282 of TRβ in an individual with a mild clinical and biochemical phenotype. Functional analyses of mutant TR using transient gene expression experiments showed no apparent dominant-negative effects. These results indicate mildly elevated thyroid hormone levels in the proband. The F245L variant identified in our patient has not been registered in widely used global variant databases, such as Single Nucleotide Polymorphism Database and ClinVar; thus, it appears to be a novel variant in the international context. However, this variant was previously included in a Japanese review article on RTHβ [10] based on an abstract presented at a domestic academic conference. To the best of our knowledge, no functional studies on this variant have been published to date. Therefore, this is the first study to provide in vitro functional evidence supporting the pathogenicity of this variant, expanding the variation spectrum of RTHβ.

The F245L variant is located near the hinge region (amino acids 174-237) [11], adjacent to previously reported variants such as R243Q, R243W, and P247L. Functional studies of R243Q and R243W have shown that these variants retain DNA-binding and heterodimerization capabilities, but exhibit reduced transcriptional activity at low concentrations of T3. This is attributed to the impaired release of corepressors, such as NCoR, ultimately leading to decreased recruitment of coactivators and transcriptional activation [12, 13]. Notably, these functional impairments were observed even though the T3-binding affinity in vitro was not markedly reduced, suggesting that ligand-dependent corepressor release, rather than ligand affinity, plays a critical role in the pathogenesis of RTHβ in these variants [12-14]. Similarly, the P247L variant reportedly reduces T3-binding affinity to approximately 30% of that of the wild-type receptor and exhibits only low-level dominant-negative activity in transient transfection assays [15]. These observations suggest that variants within this region of the ligand-binding domain may cause mild RTHβ by disrupting T3-dependent transcriptional regulation, without necessarily exerting strong dominant-negative effects.

Another mild form of RTHβ, the R316C variant, has been reported in a Japanese family with occasional nonsuppressed TSH levels and fluctuating TFT results within the normal range [16]. Functional characterization of this variant demonstrated a reduction in the T3-binding affinity to 38% of that of the wild-type receptor, while retaining DNA-binding and heterodimerization capabilities and showing impaired release of corepressors in the presence of T3 [17]. These features are reminiscent of other mild TRβ variants such as R243Q and R243W. Notably, a comprehensive analysis of > 400 RTHβ cases revealed no clear association between the cluster location of the variant and the severity of biochemical abnormalities [16]. Therefore, mild or subclinical phenotypes may arise from mutations in any of the 3 major TRβ ligand-binding domain clusters.

In our case, the F245L variant demonstrated mildly reduced transcriptional activity at physiological T3 concentrations but no apparent dominant-negative activity in vitro, a pattern similar to that of the neighboring variants described previously. Although in vitro systems, such as transient transfection assays, have inherent limitations and may not fully reflect physiological context of TRβ activity, including tissue-specific cofactors interactions or chromatin environment, these findings collectively suggest that F245L impairs TRβ function via a similar mechanism involving insufficient ligand-induced conformational changes and the impaired release of corepressors, contributing to the mild phenotype observed in this case.

In the present case, an RCC was detected in the anterior pituitary gland. However, to the best of our knowledge, no previous studies have described the cooccurrence of RTHβ and RCC to date. Furthermore, in this case, the RCC spontaneously regressed on follow-up MRI 3 years later, whereas the TFT results of the patient remained virtually unchanged. In addition, anterior pituitary hormone levels, including TSH, remained stable throughout the observation period. These findings suggest that RTHβ and RCC occurred independently and that RCC did not contribute to the thyroid function abnormality of the patient.

We report a case of RTHβ concurrent with RCC, harboring a novel heterozygous missense variant located within 1 of the clusters of *THRB*. This novel variant causes mild resistance to thyroid hormones, with mild clinical and biochemical phenotypes. Functional analyses of this novel variant, performed using transient gene expression experiments, showed no apparent dominant-negative activity of the mutant TR, reflecting mildly elevated thyroid hormone levels in the proband. In this case, concurrent RCC did not affect the pituitary-thyroid axis. This case highlights the importance of integrating clinical, biochemical, and molecular data to diagnose and characterize RTHβ, particularly in patients with subtle phenotypes and novel genetic variants.

## Learning Points

- RTHβ should be considered in patients with elevated serum thyroid hormone and nonsuppressed TSH levels, even in the absence of goiter or overt symptoms.
- Novel variants of the *THRB* gene may result in mild phenotypes and subtle functional impairments, highlighting the importance of integrating clinical, biochemical, and in vitro findings.
- Variants in the ligand-binding domain of TRβ, particularly cluster 3, may disrupt T3-dependent transcription without exerting dominant-negative effects.
- RCC can coexist with RTHβ and should not preclude a genetic diagnosis of RTHβ.

## Data Availability

Some datasets generated during and analyzed during the current study are not publicly available but are available from the corresponding author on reasonable request.

## References

[1] Pappa T, Refetoff S. Resistance to thyroid hormone beta: a focused review. Front Endocrinol (Lausanne). 2021;12:656551. 33868182 10.3389/fendo.2021.656551 PMC8044682

[2] Karczewski KJ, Francioli LC, Tiao G, et al The mutational constraint spectrum quantified from variation in 141,456 humans. Nature. 2020;581(7809):434‐443. 32461654 10.1038/s41586-020-2308-7 PMC7334197

[3] Choi Y, Sims GE, Murphy S, Miller JR, Chan AP. Predicting the functional effect of amino acid substitutions and indels. PLoS One. 2012;7(10):e46688. 23056405 10.1371/journal.pone.0046688 PMC3466303

[4] Ng PC, Henikoff S. SIFT: predicting amino acid changes that affect protein function. Nucleic Acids Res. 2003;31(13):3812‐3814. 12824425 10.1093/nar/gkg509 PMC168916

[5] Adzhubei IA, Schmidt S, Peshkin L, et al A method and server for predicting damaging missense mutations. Nat Methods. 2010;7(4):248‐249. 20354512 10.1038/nmeth0410-248 PMC2855889

[6] Cheng J, Novati G, Pan J, et al Accurate proteome-wide missense variant effect prediction with AlphaMissense. Science. 2023;381(6664):eadg7492. 37733863 10.1126/science.adg7492

[7] Tagami T, Usui T, Shimatsu A, et al Aberrant expression of thyroid hormone receptor beta isoform may cause inappropriate secretion of TSH in a TSH-secreting pituitary adenoma. J Clin Endocrinol Metab. 2011;96(6):E948‐E952. 21430027 10.1210/jc.2010-2496

[8] Tagami T, Gu WX, Peairs PT, West BL, Jameson JL. A novel natural mutation in the thyroid hormone receptor defines a dual functional domain that exchanges nuclear receptor corepressors and coactivators. Mol Endocrinol. 1998;12(12):1888‐1902. 9849963 10.1210/mend.12.12.0201

[9] Kanda Y . Investigation of the freely available easy-to-use software ‘EZR’ for medical statistics. Bone Marrow Transplant. 2013;48(3):452‐458. 23208313 10.1038/bmt.2012.244 PMC3590441

[10] Tagami T . An overview of thyroid function tests in subjects with resistance to thyroid hormone and related disorders. Endocr J. 2021;68(5):509‐517. 33827995 10.1507/endocrj.EJ21-0059

[11] Evans RM . The steroid and thyroid hormone receptor superfamily. Science. 1988;240(4854):889‐895. 3283939 10.1126/science.3283939 PMC6159881

[12] Yagi H, Pohlenz J, Hayashi Y, Sakurai A, Refetoff S. Resistance to thyroid hormone caused by two mutant thyroid hormone receptors beta, R243Q and R243W, with marked impairment of function that cannot be explained by altered in vitro 3,5,3′-triiodothyroinine binding affinity. J Clin Endocrinol Metab. 1997;82(5):1608‐1614. 9141558 10.1210/jcem.82.5.3945

[13] Safer JD, Cohen RN, Hollenberg AN, Wondisford FE. Defective release of corepressor by hinge mutants of the thyroid hormone receptor found in patients with resistance to thyroid hormone. J Biol Chem. 1998;273(46):30175‐30182. 9804773 10.1074/jbc.273.46.30175

[14] Collingwood TN, Wagner R, Matthews CH, et al A role for helix 3 of the TRbeta ligand-binding domain in coactivator recruitment identified by characterization of a third cluster of mutations in resistance to thyroid hormone. EMBO J. 1998;17(16):4760‐4770. 9707435 10.1093/emboj/17.16.4760 PMC1170805

[15] Pohlenz J, Manders L, Sadow PM, Kansal PC, Refetoff S, Weiss RE. A novel point mutation in cluster 3 of the thyroid hormone receptor beta gene (P247L) causing mild resistance to thyroid hormone. Thyroid. 1999;9(12):1195‐1203. 10646658 10.1089/thy.1999.9.1195

[16] Ueda Y, Tagami T, Tamanaha T, et al A family of RTHβ with p.R316C mutation presenting occasional syndrome of inappropriate secretion of TSH. Endocr J. 2015;62(3):251‐260. 25502991 10.1507/endocrj.EJ14-0422

[17] Nakajima Y, Yamada M, Horiguchi K, et al Resistance to thyroid hormone due to a novel thyroid hormone receptor mutant in a patient with hypothyroidism secondary to lingual thyroid and functional characterization of the mutant receptor. Thyroid. 2010;20(8):917‐926. 20615127 10.1089/thy.2009.0389

